# An Oral Vaccine Derived from Attenuated *Salmonella* Producing Murine Cytomegalovirus M24 Protein Induces Successful Antiviral Immune Responses in Mice

**DOI:** 10.3390/vaccines14030279

**Published:** 2026-03-22

**Authors:** Yujun Liu, Hao Gong, Jiaming Zhu, Fenyong Liu

**Affiliations:** 1 School of Public Health, University of California, Berkeley, CA 94720, USA; 2 Program in Comparative Biochemistry, University of California, Berkeley, CA 94720, USA

**Keywords:** cytomegalovirus, herpesvirus, immune responses, oral vaccine, *Salmonella*, vaccine

## Abstract

**Background:** Oral gene delivery vectors, such as those derived from attenuated *Salmonella* strains, have shown great promise in oral vaccine development against various human diseases. Human cytomegalovirus (CMV) is a herpesvirus capable of affecting the global population and establishing lifelong infection. Generation of an anti-CMV vaccine is a major public health priority. **Methods:** This study reports the development of a novel weakened *Salmonella* strain, S713, and the effects of this strain as an oral vaccine candidate against murine cytomegalovirus (MCMV) infection in mice. **Results:** The weakened *Salmonella* strain S713 was attenuated in killing mice in vivo by >500,000 fold compared to a clinical strain, following intragastric instillation in animals. Mice intragastrically immunized with S713 that produced MCMV M24 protein exhibited elevated anti-MCMV mucosal IgA and serum IgG titers and enhanced anti-MCMV T cell responses. Moreover, immunization with the generated vaccine in MCMV-challenged mice not only suppressed viral replication in lungs, spleens, livers, and salivary glands but also increased animal survival. **Conclusions:** These findings demonstrate strong and effective anti-MCMV immune responses induced by the generated M24-expressing vaccine. Furthermore, our results reveal the promising capability of weakened strain S713 expressing different CMV proteins to act as oral vaccines against CMV infections and diseases.

## 1. Introduction

Human cytomegalovirus (HCMV) is a herpesvirus capable of affecting populations globally and establishing lifelong infections [1,2]. It initiates congenital infections commonly associated with neurological and mental complications [3,4]. Furthermore, HCMV inflicts devastating complications in people with immunodeficiencies, such as cancer patients and organ transplant recipients [2]. Hence, the generation of an anti-HCMV vaccine represents a major public health priority and is critical for the effective prevention of HCMV infection [5,6].

Having a narrow host range, HCMV generates progeny only in human cells [2]. Thus, infections of animals by animal CMVs, including infection of mice by murine cytomegalovirus (MCMV), have been used as models to comprehend host immune responses and test vaccine candidates against CMVs [7,8]. Numerous anti-CMV vaccine candidates with various designs, such as recombinant protein-based vaccines, subviral dense bodies or virus-like particle (VLP) vaccines, and DNA vaccines, were previously investigated in animals and humans [5,6,9]. Some vaccines, including those based on attenuated *Salmonella* strains expressing MCMV antigens, produced effective anti-MCMV immunity in mice [10,11,12,13,14,15,16,17,18], implicating MCMV-infected mice as an excellent model for evaluating anti-CMV vaccine candidates. Furthermore, self-amplifying RNA- and mRNA-based vaccines have been explored against HCMV and other viruses [19,20,21]. Despite much progress on anti-HCMV vaccine research [5,6], an FDA-approved vaccine is not presently available yet against HCMV infections.

Vaccine delivery via the oral route has clear advantages compared to delivery via injection. Engineered *Salmonella* strains are promising oral gene transfer vectors for gene delivery and vaccinations [22,23,24]. Upon bacterial infection, the transgene-containing plasmids that the bacteria harbor are released as a result of the bacterial lysis inside the cells, resulting in the delivery and expression of the transgene [23]. Deactivation of *Salmonella* virulence factors, crucial for bacterial pathogenesis and intracellular infection, has been shown to increase the gene transfer ability of the constructed attenuated vector strains and reduce their virulence, achieving better vector profiles for vaccine delivery [25,26,27,28].

Previously, we have shown efficient oral delivery of antiviral ribozymes in cell cultures and in mice using engineered *Salmonella* mutant strains [28]. In the current study, we created a new weakened *Salmonella* Typhimurium strain, S713, from SL7207, an auxotrophic aroA mutant [29], by removing the gene encoding bacterial small non-coding RNA (sRNA) IsrM. IsrM is a virulence factor critical for virulence and colonization in mice and intracellular replication inside macrophages [30]. We report here the generation of a *Salmonella*-based vaccine, Vac-M24, by the transformation of S713 with a plasmid construct for the expression of MCMV M24 protein.

HCMV and MCMV are the largest human and murine herpesvirus, respectively [2,7]. However, many ORFs encoded by HCMV and MCMV have not been studied as antigens for vaccine development. M24 and its HCMV homolog, UL24, are structural components found in infectious virions, and therefore may serve as ideal antigens for the development of anti-CMV vaccines [2,31]. While little is known about the function of M24 in MCMV infection, our previous study showed that a UL24-deletion HCMV mutant grew well in human fibroblasts but was attenuated in growth in human microvascular endothelial cells, suggesting that UL24 encodes a tropism factor important for viral replication in endothelial cells [32]. In recent studies, UL24 has been shown to be involved in modulating the cellular localization of a host restriction factor and regulating the expression of an immunoregulatory protein [33,34]. These observations highlight the important roles of UL24 and potentially M24 in viral pathogenesis and viral-host interactions in vivo. However, little is known about M24-specific immunity or T cell-mediated host defense in MCMV-infected mice, while UL24 appeared to have the potential to induce antiviral CD4 and CD8 T cells but did not emerge as predominant immunogens in humans naturally infected with HCMV [35]. It is currently not known if M24 or UL24 are employed as antigens for anti-CMV vaccination.

This current study shows that a weakened *Salmonella* vector containing the M24 expression cassette, when orally administered in mice, produced robust anti-MCMV immune responses. Moreover, immunization with the generated vaccine in MCMV-challenged mice not only suppressed viral replication but also increased animal survival. These findings reveal the capability of the weakened strain S713 in expressing different CMV proteins to act as promising oral vaccines against CMV infections and diseases.

## 2. Materials and Methods

### 2.1. Cells, Salmonella, Viruses, and Plasmid Constructs

MCMV (Smith) was obtained from American Type Culture Collection (ATCC, Manassas, VA, USA) [16,36]. Following the BAC-mid approach [32,37], we created mutant m-M24, in which the M24 open reading frame (ORF) sequence (coordinates 25151–26119) was deleted from the MCMV genome (accession number U68299) [38]. The genomic DNA of viral mutant m-M24 was examined by restriction digestion and sequencing analysis experiments to confirm the M24 ORF deletion. The MCMV Smith strain and m-M24 mutant were propagated in NIH 3T3 cells following our previously described protocols [16,36].

The DNA sequence for M24 ORF was produced by PCR with MCMV (Smith) DNA as the template using 5′ primer 5M24 (5′-GGGAATTCCATATGAAGATCTCTGATTCGACGGGGAAAATCG-3′) and 3′ primer 3M24 (5′-CCGGAATTCGGTACCCTAGAACTCGCTGAGGCG-3′). The M24 ORF sequence was then inserted into expression vector pV100 to develop the M24 expression plasmid pV-M24. Construct pV100 is a derivative of pVAX (Invitrogen, Carlsbad, CA, USA), with additional restriction enzyme cloning sites, a kanamycin resistance gene for selection in *E. coli*, and a CMV major immediate-early gene promoter for expression in mammalian cells.

*Salmonella* Typhimurium strain SL7207 (an *aroA* mutant from Bruce A. D. Stocker, Stanford University, CA) [29] and clinical isolate ST14028s were documented in previous studies [30,39]. Using the λ Red recombinase-based mutagenesis procedure [40], attenuated strain S713 was created by deleting the sequence coding for bacterial sRNA IsrM, following our previously described protocols [30]. In the experiments, PCR products were generated by employing PCR to amplify the kanamycin resistance gene sequence with plasmid construct pKD4 as the template using primers P5ΔIsrM (5′-TATCAAGCCTTTATCATTTTAAACTGAATTACGGTAGGCGTACCAACTTTGTATAAGATACATATGAATATCCTCCTTAGTTC-3′) and P3ΔIsrM (5′-CTCATTCAGGGTGCCATAACTCGTAGTTCTCAGCAATTCTCACTGGACGACAATAGACGTTGTGTAGGCTGGAGCTGCTT-3′) and then transformed into SL7207-carrying plasmid construct pKD46. Employing PCR, we verified the deletion with primers IsrM-5P (5′-TGTCCATTTAGTCACCATTACT-3′) and IsrM-3P (5′-AGCTTCTTAGCGATTTTTGCCA-3′). We conducted phage P22 transduction to transfer the resulting deletion to a new SL7207 culture to produce the non-polar strain S713. The IsrM deletion in S713 was verified by PCR and sequencing analysis [30,39,40].

### 2.2. Construction and Characterization of the Vaccines

We created vaccines Vac-C and Vac-M24 by transforming empty vector pV100 and M24 expression plasmid pV-M24 into strain S713, respectively. We analyzed bacterial growth in vitro in LB broth [30,39] and the results were the means and standard deviations from three independent experiments, each of which was in duplicate.

*Salmonella*-based vaccines Vac-M24 and Vac-C were incubated with mouse J774 macrophages (ATCC) to allow infection in order to assess gene transfer and M24 expression, following previously described protocols [28]. At 3 days postinfection, proteins from cells were run on SDS-containing polyacrylamide gels, electrically transferred, mixed with a rabbit anti-M24 polyclonal antibody (Promab, Inc, Richmond, CA, USA), stained with an antibody coupled with the ECL substrates (Thermo Fisher Scientific, Waltham, MA, USA), and finally imaged employing a STORM 840 Phosphorimager [28,30]. The capability of *Salmonella* to kill mice was evaluated by the intragastric inoculation of groups of five-week-old BALB/c mice (Jackson Laboratory, Bar Harbor, ME) (5 per group) with different bacteria (2 × 10^3^ cfu of *Salmonella* ST14028 per mice or 1 × 10^9^ cfu of SL7207, Vac-C, and Vac-M24 per mice) for up to 60 days postinfection. We examined and recorded animal survival daily.

### 2.3. Oral Vaccination and Administration

We anesthetized groups of five-week-old BALB/c mice (Jackson Laboratory, Bar Harbor, ME) (5 mice per group) with isoflurane and intragastrically administered them with phosphate-buffered saline (PBS) only, or PBS with 1 × 10^9^ cfu Vac-C or Vac-M24 [30,39]. We administered the PBS or vaccines to the mice three times on day 0, 14, and 28. Two trials with 5 mice per group were executed for each experiment.

### 2.4. Antibody Responses Assayed by ELISA and T Cell Responses by ELISPOT Assay

Antigen samples used in both ELISA and ELISPOT assays included lysates from NIH 3T3 cells infected with the MCMV Smith strain and its mutant m-M24, which contained the M24 ORF deletion. These lysates were produced by harvesting and processing Smith-infected or m-M24-infected cells (MOI = 1) at 96 h, following our previously described protocols [36].

We immunized five-week-old BALB/c mice with PBS only, Vac-C, and Vac-M24 at days 0, 14, and 28, and collected splenocytes, mucosal wash, and serum samples at two weeks after the third immunization [41,42]. In the ELISA assay to obtain IgG and IgA titers that reacted with Smith-infected or m-M24-infected cell lysates, Medisorp plates (Thermo Fisher, Waltham, MA, USA) were incubated with cell lysates, then mixed with nasal wash or serum, reacted with anti-mouse IgG- or IgA-AP secondary antibodies (Cell Signaling Technologies, Danvers, MA, USA), stained with a BioLegend chemiluminescent substrate (San Diego, CA, USA), and evaluated in a Spectramax instrument (San Jose, CA, USA). Each assay was executed in duplicate and repeated three times.

To evaluate the levels of T cells producing IFN-γ [43], IFN-γ ELISPOT plates (U-Cytech biosciences, Utrecht, The Netherlands) were initially mixed with collected splenocytes (1 × 10^6^ cells) and Smith- or m-M24-infected cell lysates. The plates were then allowed to bind to anti-IFN-γ antibodies and quantified for IFN-γ-producing T cell numbers [43]. Each assay was executed in duplicate and repeated three times.

### 2.5. Assaying Animal Survival and Virus Growth in Vaccinated Mice Challenged with MCMV

We administered PBS or vaccines to five-week-old BALB/c mice (5–10 animals per group) three times on day 0, 14, and 28. At day 42 (two weeks after the final administration). Each mouse was intranasally or intraperitoneally infected with 1 × 10^6^ PFU MCMV (Smith) that were passaged in the salivary glands of BALB/c mice in lethal dosage challenge experiments, or 5 × 10^4^ PFU MCMV (Smith) that were passaged in cultured NIH 3T3 cells in sublethal dosage challenge experiments [36]. We observed those animals with MCMV lethal dosage challenge for 14 days and recorded their mortalities.

At 120 h (5 days) post-challenge, lungs, livers, spleens, and salivary glands were obtained from animals with MCMV sublethal dosage challenge. The amounts of virus in these samples were uncovered by the plaque assays as described [36]. Each assay was executed in duplicate and repeated three times.

### 2.6. Statistical Analysis

Each experiment, in duplicate, was reiterated three times. Analysis of variance (ANOVA) was executed for statistical analyses using GraphPad Prism software (version 10) (San Diego, CA, USA). A *p*-value of <0.05 was viewed as significant.

## 3. Results

### 3.1. Creation and Characterization of Salmonella-Based Vaccines

We previously successfully employed attenuated *Salmonella* as oral gene transfer vectors for expressing antiviral ribozymes in vitro and in vivo [28]. In the current study, we produced a new attenuated strain, S713, from *Salmonella* Typhimurium strain SL7207 [29] by removing the gene encoding the bacterial small non-coding RNA (sRNA), IsrM. IsrM, a pathogenicity island-encoded sRNA, has been shown to be crucial for bacterial virulence and colonization in mice and intracellular replication inside macrophages [30]. IsrM targets the mRNAs coding for SopA, an effector of *Salmonella* pathogenicity island 1 (SPI-1) and HilE, a global regulator of the expression of SPI-1 proteins. The deactivation of IsrM reduces bacterial virulence in mice and intracellular replication/survival in macrophages [30]. Because of the inactivation of IsrM, we believe the further attenuation of strain S713 in virulence and pathogenicity compared to SL7207.

We produced two *Salmonella*-based vaccines from the newly constructed bacterial strain S713. We created the functional vaccine, Vac-M24, by transforming into S713 the construct pV-M24, which contained the expression cassette for the MCMV M24 ORF sequence driven by a eukaryotic promoter. We also created a control vaccine, Vac-C, by introducing into S713 the DNA of the empty vector pV100 without the M24 sequence.

**Figure 1:**
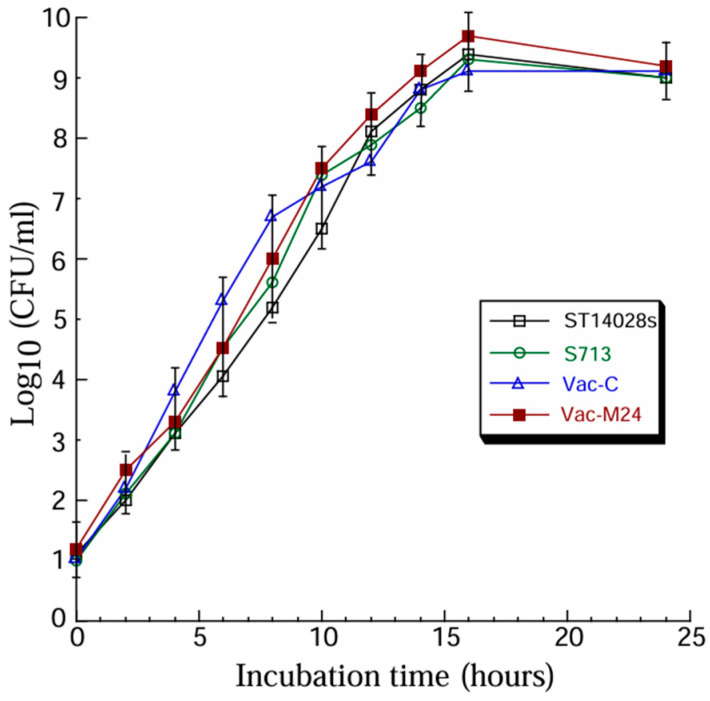
*Salmonella* in vitro growth in LB broth. Results are the means of three experiments, each of which was executed in duplicate. The error bars denote the standard deviations.

Experiments were executed to examine the produced vaccines in vitro and in vivo in mice. First, we examined the growth of the constructed vaccines in vitro in LB broth. We found no remarkable differences in growth among Vac-C, Vac-M24, the parental strain S713, and the clinical strain ST14028s (Figure 1). Our findings suggest that the expression vector pV100 and M24 ORF might not interfere with the viability and growth of vaccines Vac-C and Vac-M24.

Second, we examined the expression of M24 in mouse cells upon infection with vaccine Vac-M24 that contained the M24 ORF expression cassette. In Western blot analysis experiments, we found the presence of ~40 kDa M24 protein in Vac-M24-infected macrophages (Supplementary Materials, Figure S1). We found no presence of M24 protein in cells infected with Vac-C, which contained no M24 ORF. Moreover, we found no presence of M24 protein when vaccine Vac-M24 was cultured alone in LB broth, expected due to the control of M24 expression cassette by a eukaryotic promoter from the expression plasmid pV-M24 of this vaccine, which was only turned on in mammalian cells such as J774.

**Figure 2:**
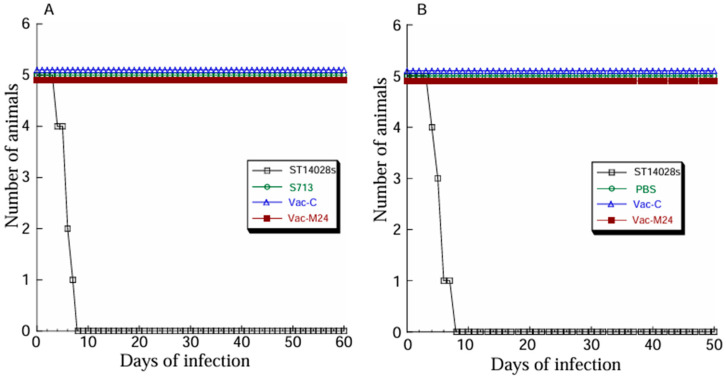
(**A**) Animal mortality after administration with 2 × 10^3^ CFU of *Salmonella* ST14028 per mice, or 1 × 10^9^ CFU of SL7207, Vac-C, and Vac-M24 per mice. (**B**) Animal mortality after administration with 2 × 10^3^ CFU of *Salmonella* ST14028 per mice once on day 0 or with phosphate-buffered saline (PBS), 1 × 10^9^ CFU of Vac-C, and Vac-M24 per mice three times on days 0, 14, and 28. BALB/c mice (group of 5 animals) were intragastrically administered with either PBS or *Salmonella* and examined for their mortality until 50 (**B**) or 60 days (**A**) post-administration.

Third, we examined the capability of vaccines Vac-C and Vac-M24 to kill mice. We found that all mice died within 7 days after intragastric inoculation with a 2 × 10^3^ cfu/mouse dose of ST148028s, which is a clinical strain with known virulence in killing mice in vivo (Figure 2A). Even at 60 days post-inoculation, however, we did not find any deaths of mice that were inoculated with a higher dose (1 × 10^9^ cfu/mouse) of Vac-C, Vac-M24, and S713 (Figure 2A). Additional experiments further showed no death of mice that were intragastrically administered with Vac-C (1 × 10^9^ cfu per mice per administration), Vac-M24 (1 × 10^9^ cfu per mouse per administration), and PBS, three times at days 0, 14, and 28 in the 50 days post-administration (Figure 2B). All these animals exhibited no substantial weight changes during the time course. Within 7 days post-administration, however, we found that all animals died after intragastrical administration with a 2 × 10^3^ cfu/mouse dose of ST148028s, a known virulent clinical strain (Figure 2B). These findings indicate that Vac-M24 and Vac-C were considerably attenuated in killing mice in vivo.

### 3.2. Anti-MCMV Humoral Responses Induced by Vac-M24 Vaccination

Our immunization protocols involved the intragastrical administration of animals (5 mice per group) using PBS only or Vac-C (1 × 10^9^ cfu per mice per administration) and Vac-M24 (1 × 10^9^ cfu per mice per administration) on day 0, 14, and 28. These animals stayed healthy up to 42 days post-administration when we end the procedures 14 days after the third administration (Figure 2B). Serum and nasal wash mucosal samples were collected while splenocytes were isolated from the spleens collected from the animals at 42 days post-administration.

To evaluate IgG humoral responses, we employed ELISA assays to examine the presence of anti-MCMV antibodies in the serum samples collected from the vaccinated mice. The antigens included the lysate samples from cells infected with the MCMV wildtype Smith strain and those infected with mutant m-M24. Mutant m-M24, constructed from the Smith strain by the deletion of the M24 ORF sequence, was found to replicate in NIH 3T3 cells as well as the Smith strain. Because this mutant lacked the M24 ORF sequence, we used the m-24 infected cell lysates to evaluate the specificity of the elicited antibody responses against M24 protein.

**Figure 3:**
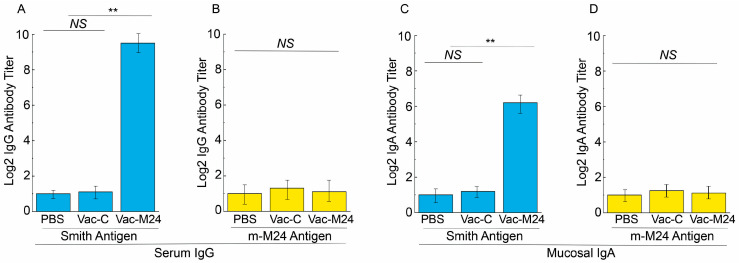
Anti-MCMV serum IgG (**A**,**B**) and mucosal IgA levels (**C**,**D**) from the intragastrically immunized animals. We immunized mice (*n* = 5 per group) with PBS only, Vac-C, and Vac-M24 at days 0, 14, and 28, and collected mucosal wash and serum samples at two weeks after the third immunization. Smith-infected (**A**,**C**) and m-M24-infected cell lysates (**B**,**D**) were applied for the ELISA assays. ** *p* < 0.05. NS, not significant. Results are the means of three experiments, each of which was executed in duplicate. The error bars denote the standard deviations.

Sera from Vac-M24-vaccinated animals elicited about 350 times higher antibody titers against the Smith-infected cell lysates than sera from the Vac-C- and PBS-administered mice (Figure 3A). However, these same sera from the Vac-M24 administered mice had noticeably low and comparable humoral responses against the m-M24-infected cell lysates as those from the Vac-C- and PBS-administered mice (Figure 3B). These findings imply that the functional vaccine Vac-M24 produces strong anti-MCMV M24-specific IgG antibody responses.

Experiments were also executed to evaluate the mucosal humoral responses produced by the vaccines since *Salmonella*, a pathogen colonizing in mucosal sites such as the gastrointestinal track, can efficiently generate mucosal immune responses [44]. To evaluate mucosal IgA responses, we employed ELISA assays to examine the presence of anti-MCMV antibodies in the nasal wash samples collected from the vaccinated mice.

The nasal wash samples from Vac-M24-vaccinated animals had about 45-fold higher IgA titers against the Smith-infected cell lysates than those from the Vac-C- and PBS-administered mice (Figure 3C). However, these same nasal wash samples from the Vac-M24 administered mice had noticeably low and comparable humoral responses against the m-M24-infected cell lysates as those from the Vac-C- and PBS-administered mice (Figure 3D). These findings imply that the functional vaccine Vac-M24 also produces anti-MCMV M24-specific mucosal IgA responses.

**Figure 4:**
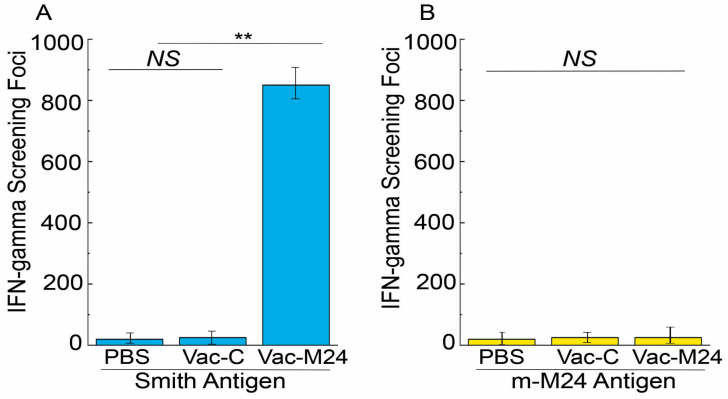
Anti-MCMV T cell response levels from the intragastrically immunized animals. We immunized mice (*n* = 5 per group) with PBS only, Vac-C, and Vac-M24 at days 0, 14, and 28, and collected splenocytes at two weeks after the third immunization. Smith-infected (**A**) and m-M24-infected cell lysates (**B**) were applied for the ELISPOT assays, which quantified IFN-γ-producing T cells by computing the number of spot-forming cells (SFC) per 1 × 10^6^ cells. ** *p* < 0.05. NS, not significant. Results are the means of three experiments, each of which was executed in duplicate. The error bars denote the standard deviations.

We employed ELISPOT assays to evaluate the vaccine-induced T cell responses. Splenocytes from mice immunized with Vac-M24 had about 80 times more IFN-γ-producing T cells against the Smith-infected cell lysates than those from the Vac-C- and PBS-administered mice (Figure 4A). However, these splenocytes from the Vac-M24 administered mice had noticeably low and comparable numbers of IFN-γ-producing T cells against the m-M24-infected cell lysates as those from the Vac-C- and PBS-administered mice (Figure 4B). These findings imply that the functional vaccine Vac-M24 produces strong anti-MCMV M24-specific T cell responses.

### 3.3. Antiviral Immune Protection Against MCMV Challenge by Vac-M24 Vaccination

The first series of experiments was to evaluate the levels of anti-MCMV immune protection against MCMV systemic and mucosal challenge provided by Vac-M24 vaccination. Our experiment protocols involved intragastrical administration of groups of mice using PBS or Vac-C (1 × 10^9^ cfu per mice per administration) and Vac-M24 (1 × 10^9^ cfu per mice per administration) at days 0, 14, and 28. Forty-two days after the first administration, these mice were subjected to intraperitoneal or intranasal challenge using highly pathogenic salivary gland-passaged MCMV.

**Figure 5:**
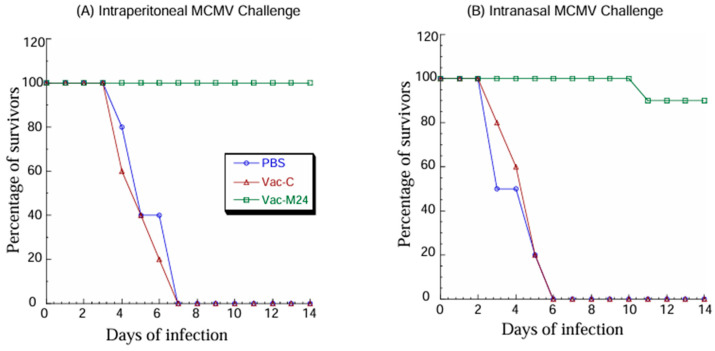
Mortality of vaccinated animals after intraperitoneal (**A**) and intranasal (**B**) MCMV challenge. BALB/c mice intragastrically administered with PBS only, Vac-C, and Vac-M24 three times at days 0, 14, and 28 were challenged with 1 × 10^6^ PFU salivary gland-passaged MCMV per mice at two weeks after the third immunization. Their mortality was examined daily and recorded.

When subjected to intraperitoneal MCMV challenge, none (0%) of mice vaccinated with Vac-M24 died at 14 days post-challenge while all (100%) of the mice with PBS and Vac-C died within 7 days post-challenge (Figure 5A). When subjected to intranasal MCMV challenge, only 10% of mice vaccinated with Vac-M24 died at 14 days post-challenge while all (100%) of mice with PBS and Vac-C died within 6 days post-challenge (Figure 5B). All the surviving animals exhibited no substantial weight changes during the time course. These findings suggested that the Vac-M24 vaccination provided 100% and 90% immune protection against systemic and mucosal MCMV challenge, respectively, while the control vaccine Vac-C did not provide any immune protection.

The second series of experiments was to determine if Vac-M24 vaccination reduces MCMV infection and growth after virus systemic and mucosal challenge. Our experiment protocols involved the intragastrical administration of groups of mice with PBS or Vac-C (1 × 10^9^ cfu per mice per administration) and Vac-M24 (1 × 10^9^ cfu per mice per administration) at days 0, 14, and 28. Forty-two days after the first administration, these mice were subjected to intraperitoneal or intranasal challenge using sublethal doses of MCMV. Animals were sacrificed at 5 days (120 h) post-challenge and the amounts of virus in lungs, livers, spleens, and salivary glands were obtained to evaluate the effect of vaccination on MCMV infection and growth.

**Figure 6:**
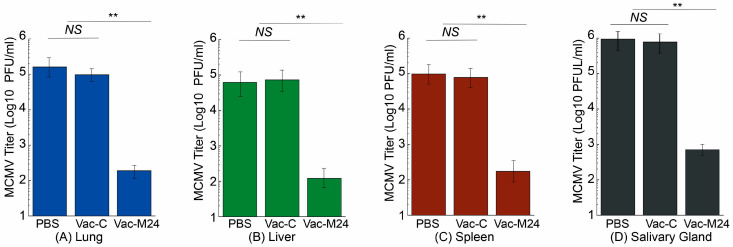
MCMV levels in the intraperitoneally challenged animals. BALB/c mice intragastrically administered with PBS only, Vac-C, and Vac-M24 three times at days 0, 14, and 28 were intraperitoneally challenged with 5 × 10^4^ PFU MCMV per mice at two weeks after the third immunization. Five days (120 h) after challenge, the amounts of MCMV in the lungs (**A**), livers (**B**), spleens (**C**), and salivary glands (**D**) were quantified with plaque assays. ** *p* < 0.05. NS, not significant. Results are the means of three experiments, each of which was executed in duplicate. The error bars denote the standard deviations. The limit of virus detection in the organ homogenates was 10 PFU/mL of the sonicated mixture.

**Figure 7:**
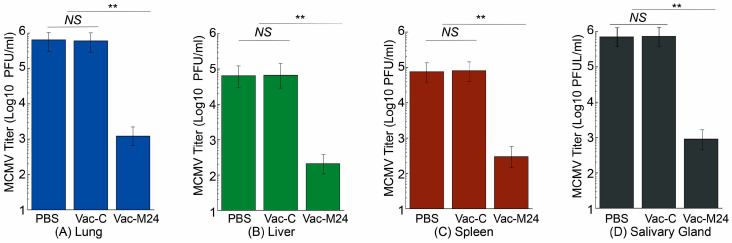
MCMV levels in the intranasally challenged animals. BALB/c mice intragastrically administered with PBS only, Vac-C, and Vac-M24 three times at days 0, 14, and 28 were intranasally challenged with 5 × 10^4^ PFU MCMV per mice at two weeks after the third immunization. Five days (120 h) after challenge, the amounts of MCMV in the lungs (**A**), livers (**B**), spleens (**C**), and salivary glands (**D**) were quantified with plaque assays. ** *p* < 0.05. NS, not significant. Results are the means of three experiments, each of which was executed in duplicate. The error bars denote the standard deviations. The limit of virus detection in the organ homogenates was 10 PFU/mL of the sonicated mixture.

In experiments with intraperitoneal MCMV challenge, Vac-M24-vaccinated animals exhibited 800, 500, 550, and 1200-fold lower MCMV titers in the lungs, livers, spleens, and salivary glands than PBS-administered animals, respectively (Figure 6). Vac-C-vaccinated animals, however, exhibited noticeably high and comparable MCMV titers in these organs to the PBS-administered animals. Similarly, in experiments with intranasal MCMV challenge, Vac-M24-vaccinated animals exhibited 550, 300, 250, and 800-fold lower MCMV titers in the lungs, livers, spleens, and salivary glands than PBS-administered animals (Figure 7). Vac-C-vaccinated animals, however, exhibited noticeably high and comparable MCMV titers in these organs to the PBS-administered animals. Our findings indicated that vaccination of Vac-M24 inhibited viral infection and growth in mice subjected to both intraperitoneal and intranasal MCMV challenge.

## 4. Discussion

Generation of an anti-human CMV vaccine is globally important for public health due to the ubiquitous prevalence and lifelong pathogenesis of the HCMV infection [5,6]. Causing common congenital infections, HCMV is associated with devastating complications and is a tremendous economic burden to the society [2]. An effective vaccine is much needed to control and prevent HCMV infection and its associated diseases. However, an FDA-approved anti-HCMV vaccine is not yet presently available [5,6].

Vaccine delivery via an oral route by *Salmonella*-based vectors has a clear advantage compared to vaccine delivery via injection. In the current study, we created a new weakened strain, S713, from the aroA mutant SL7207 [29] by removing the gene encoding bacterial small non-coding RNA (sRNA), IsrM. IsrM is a virulence factor critical for bacterial virulence and colonization in mice and intracellular survival in macrophages [30]. IsrM deactivation is known to reduce bacterial virulence in mice and intracellular replication/survival in macrophages [30].

Because of the IsrM deletion mutation, we believe further attenuation of strain S713 in virulence and pathogenicity with reduced intracellular survival and increased transgene delivery efficiency. Our intent is to develop S713 as a potential oral gene transfer vector for gene therapy and vaccine applications. Indeed, compared to strain ST14802s, a known virulent clinical isolate, S713 and its derived vaccines Vac-M24 and Vac-C were remarkably attenuated to kill mice (Figure 2). Compared to ST14802s, Vac-M24, which possessed the M24 expression cassette, was not deficient in viability and replication when grown in vitro in LB (Figure 1) and was able to deliver the M24 ORF sequence for expression in mouse cells. These observations reveal that the new strain S713 and its derived oral vaccines retain their ability to grow and replicate as the *Salmonella* clinical strain in vitro and possess the capability for gene transfer to express antigens in cells. It will be interesting to compare the intracellular survival and transgene delivery efficiency between S713 and its parental strain SL7207 to understand the ability of S713 as a delivery vector.

Compared to Vac-C or PBS administration, vaccination with Vac-M24 in mice increased anti-MCMV serum IgG and mucosa IgA levels and elevated T cell responses specifically against M24 (Figure 3 and Figure 4). Moreover, compared to Vac-C or PBS administrations, vaccination with Vac-M24 reduced viral growth in the spleens, livers, lungs, and salivary glands and decreased animal mortality in mice that were subjected to intraperitoneal and intranasal MCMV challenge (Figure 5, Figure 6 and Figure 7). These findings showed that oral vector S713 expressing the M24 antigen produced effective anti-MCMV immune protection and responses in immunized mice. Moreover, our findings demonstrate the capability of the weakened strain S713 as a new oral vector for generating innovative vaccines again CMV infections and diseases.

Vaccination with live bacteria such as *Salmonella* poses potential safety concerns such as pathological consequences or effects associated with ongoing bacterial infection. An FDA-approved live *Salmonella*-based vaccine, called Ty21a, has been used in humans for the prevention of typhoid fever [45,46,47]. This vaccine has been shown to elicit cellular, local, and systemic immunity at the same time [48,49]. Vaccine Ty21a, based on an attenuated *Salmonella typhi* strain, is effective against typhoid fever in more than 50% of the population [45,46,47]. These results illustrate that weakened strains may be safe and effective as oral vaccine vectors for vaccination in humans [50].

Nonetheless, extra investigations should be performed to address side effects and safety concerns due to the use of live bacteria. We used a dose of 1 × 10^9^ CFU bacteria for vaccination per mice to ensure maximal delivery of the M24 construct for efficient antigen expression. Further studies of different dose titrations for vaccination may be carried out to determine the optional doses for vaccination to generate efficient M24 expression and induce effective antiviral immunity. It would be ideal to use the lowest dose of bacteria for vaccination that yields the highest efficiency of gene transfer and produce the strongest immune responses. Other issues for consideration include the clearance and turnover of the bacteria after oral administration and vaccination. The delivery mechanism of transgenes for expression by attenuated *Salmonella* is currently not completely understood, although the intracellular survival and replication of *Salmonella* has been extensively studied. *Salmonella* encodes many virulence determinants in supporting intracellular survival and counteracting both innate and adaptive immune responses [22,23,24]. Creation and engineering of new strains deactivating or removing virulence determinants may potentially lead to better live-attenuated vectors with reduced virulence and pathogenicity, decreased intracellular survival, and increased clearance in the host.

Several candidates with various anti-CMV vaccine designs, such as DNA-based, self-amplifying RNA- and mRNA-based, recombinant protein-based, and live attenuated virus vector-based vaccines, were previously investigated [5,6,9,19,20,21], and some produced effective anti-MCMV immunity in mice [10,11,12,13,14]. In particular, vaccination with a *Salmonella*-based vaccine harboring a full length MCMV genome induced anti-MCMV antibody responses and protected mice from MCMV lethal challenge [18]. HCMV and MCMV are the largest human and murine herpesvirus, respectively [2,7]. However, many ORFs encoded by HCMV and MCMV have not been studied as antigens for vaccine development. Whether many of these viral antigens induce effective antiviral immunity in vaccinated animals and humans have not been studied. We have recently constructed *Salmonella* strains that contained different mutations and expressed numerous MCMV antigens, such as M25, M33, and M78 proteins, and showed that these strains could function as vaccine candidates in eliciting strong anti-MCMV immunity in vaccinated mice [15,16,17]. The vaccine used in this study, Vac-M24, was derived from S713, a novel strain with IsrM deletion, and expressed M24, an antigen that has not been evaluated in previous vaccine research. Collectively, these studies showed the promise of using attenuated *Salmonella* strains carrying viral antigens as vaccine candidates against MCMV and potentially HCMV.

As structural components found in the infectious virions, M24 and UL24 are considered ideal antigens for the development of anti-CMV vaccines [2,31]. Viral mutants with the deletion of UL24 and M24 can grow in cultured cells, indicating that these two genes are not essential for viral replication in vitro. While the function of M24 in MCMV infection has not been investigated extensively, our previous study showed that a UL24-deletion HCMV mutant grew well in human fibroblasts but was attenuated in growth in human microvascular endothelial cells, suggesting that UL24 encodes a tropism factor important for viral replication in endothelial cells [32]. In recent studies, UL24 has been shown to cooperate with UL43 protein to modulate the cellular localization of a host restriction factor, SAMHD1, and regulate the expression of immunoregulatory UL16 binding protein 1 (ULBP1) [33,34]. These observations highlight the important roles of UL24, and potentially M24, in viral pathogenesis and viral–host interactions in vivo. However, little is known about M24-specific T cell-mediated host defense in MCMV-infected mice, while UL24 could elicit antiviral CD4 and CD8 T cells but did not appear to be predominant immunogens in humans naturally infected with HCMV [35].

Our results showed that animals vaccinated with Vac-M24 exhibited elevated levels of anti-MCMV IgG and IgM antibody and T cell immune responses, compared to those administered with PBS or control Vac-C (Figure 3 and Figure 4). It will be interesting to perform further experiments to evaluate the immune protection significance of these antibody and T cell responses, such as by either depletion studies or adoptive transfer to examine their host defense value. These studies will provide insights into the antiviral mechanism of the immune responses induced by the constructed vaccine.

In this report, immunization with Vac-M24 produced strong T cell and humoral immunity and anti-MCMV protection against viral intraperitoneal and intranasal challenge. These observations reveal the potential of attenuated *Salmonella* expressing different CMV antigens, such as UL24, as oral vaccine candidates to prevent HCMV infection. However, whether *Salmonella*-based vaccines expressing UL24 can also induce effective anti-HCMV immunity in humans needs to be investigated. Further studies on different designs of *Salmonella*-based vectors and choice of CMV antigens will assist our efforts for anti-human CMV oral vaccine development.

## 5. Conclusions

HCMV is a herpesvirus affecting the global population with lifelong infection. Generation of an anti-HCMV vaccine represents a major public health priority. This study produced a novel weakened *Salmonella* strain, S713, and investigated the effects of this strain as an oral vaccine candidate against murine cytomegalovirus (MCMV) infection in mice. Our results showed that S713 was remarkably attenuated in killing mice in vivo. Oral vaccination with S713-producing MCMV M24 protein induced mucosal IgA and serum IgG titers and T cell responses against MCMV in animals. Furthermore, vaccination of S713-expressing M24 protein in MCMV-challenged mice suppressed viral replication in mice and increased animal survival. These findings show the potential of the weakened strain S713 expressing different viral proteins to act as promising oral vaccines against cytomegalovirus infections and diseases.

## Data Availability

The dataset is available on request from the authors.

## Supplementary Materials

The following supporting information can be downloaded at: https://www.mdpi.com/article/10.3390/vaccines14030279/s1, Figure S1: Western blot detection of M24 expression.
