# Supplementary figures and images for: An Oral Vaccine Derived from Attenuated Salmonella Producing Murine Cytomegalovirus M24 Protein Induces Successful Antiviral Immune Responses in Mice
Source: Vaccines (Basel). 2026 Mar 22;14(3):279. doi: 10.3390/vaccines14030279 (PMC13030467; doi:10.3390/vaccines14030279)

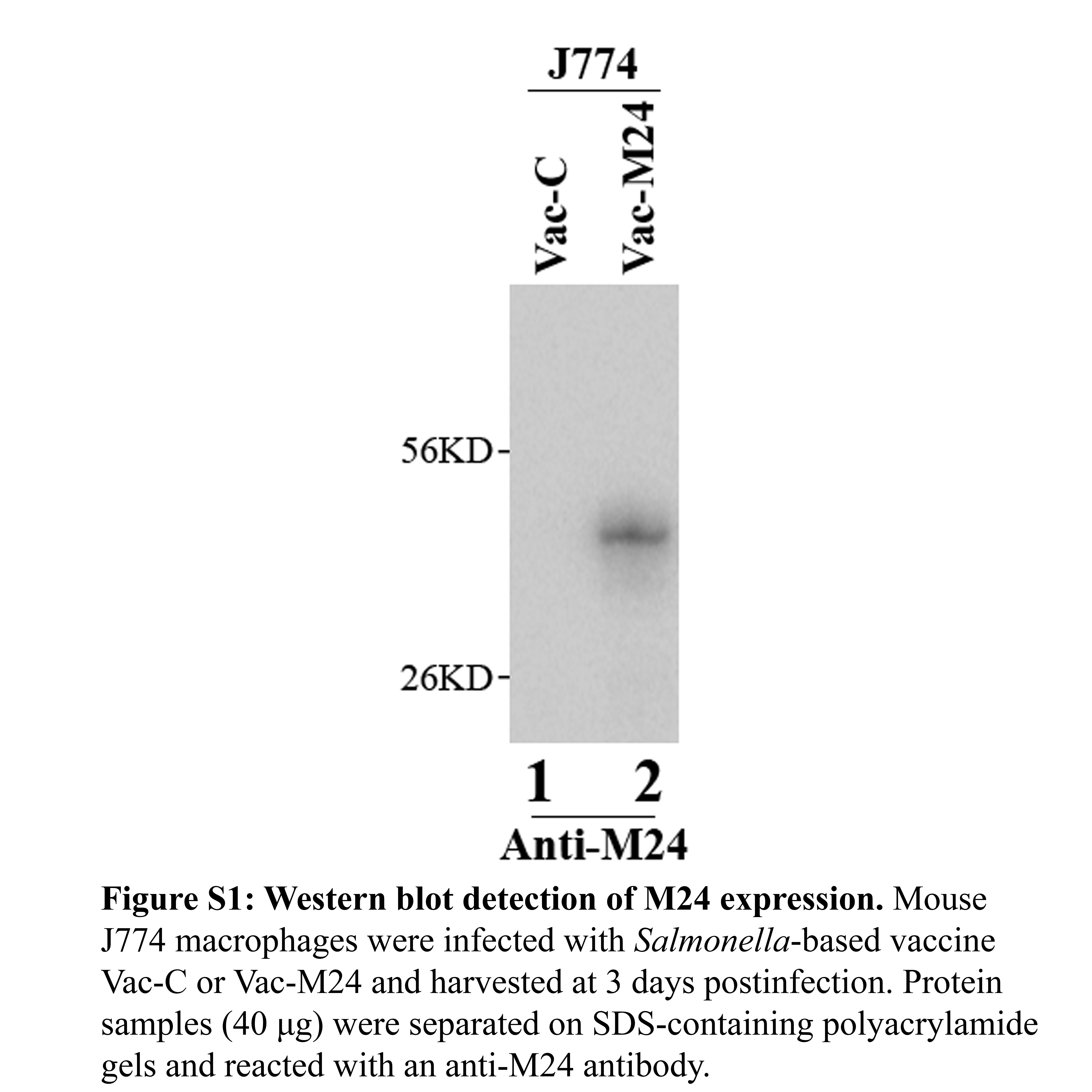

Supplement: Supplementary file 1 [file vaccines-14-00279-s001.zip › vaccines-3975712-supplementary.jpg]
